# Prevalence, Patterns, and Clinical Predictors of Left Ventricular Late Gadolinium Enhancement in Patients Undergoing Cardiac Magnetic Resonance Prior to Pulmonary Vein Antral Isolation for Atrial Fibrillation: A Cross-Sectional Observational Study

**DOI:** 10.1097/MD.0000000000001384

**Published:** 2015-09-18

**Authors:** John W. Nance, Irfan M. Khurram, Saman Nazarian, Jane DeWire, Hugh Calkins, Stefan L. Zimmerman

**Affiliations:** From the Department of Radiology (JWNJ, SLZ); and Department of Medicine/Cardiology, Johns Hopkins Medical Institutions, Baltimore, MD (IMK, SN, JD, HC).

## Abstract

Cardiac magnetic resonance (CMR) imaging is increasingly used to evaluate patients with atrial fibrillation (AF) before pulmonary vein antral isolation (PVAI). The purpose of this study was to assess the incidence and pattern of left ventricular (LV) late gadolinium enhancement (LGE) in patients undergoing CMR before PVAI and compare the clinical and demographic differences of patients with and without LV LGE.

Clinical and demographic data on 62 patients (mean age 61 ± 7.9, 69% male) undergoing CMR before PVAI for AF were collected. Two observers, masked to clinical histories, independently recorded the prevalence, extent (number of myocardial segments), and pattern (subendocardial, midmyocardial, or subepicardial) of LV LGE in each patient. Clinical and demographic predictors of LV LGE were determined using logistic regression.

Twenty-three patients (37%) demonstrated LV LGE affecting a mean of 3.0 ± 2.1 myocardial segments. There was no difference in LV ejection fraction between patients with and without LGE, and most (65%) patients with LGE had normal wall motion. Only age (*P* = 0.04) and a history of congestive heart failure (*P* = .03) were statistically significant independent predictors of LGE. The most common LGE pattern was midmyocardial, seen in 17 of 23 (74%) patients. Only 4 of 23 (17%) patients had LGE in an “expected” pattern based on clinical history. Of the remaining 19 patients, 4 had known congestive heart failure, 5 nonischemic cardiomyopathy, 4 known coronary artery disease, and 2 prior aortic valve replacement. Six of 23 (26%) patients had no known coronary artery, valvular, or myocardial disease.

There is a high prevalence of unexpected LV scar in patients undergoing CMR before PVAI for AF, with most patients demonstrating a nonischemic pattern of LV LGE and no wall motion abnormalities (ie, subclinical disease). The high prevalence of unexpected LGE in these patients may argue for CMR as the modality of choice for imaging integration before PVAI, especially given the demonstrated prognostic value of LGE in this and other patient populations.

## INTRODUCTION

Atrial fibrillation (AF) is a common clinical problem, affecting an estimated 2.3 million adults in the United States in 2011, with a projected rise in prevalence to 5.6 million by the year 2050.^1^ Pulmonary vein antral isolation (PVAI) via endovascular catheter ablation has been validated as an effective therapy for symptomatic AF, with evidence supporting the procedure both in patients refractory to/intolerant of antiarrhythmic medications and in select patients before the initiation of medical antiarrhythmic therapy.^2–4^ Although PVAI can be performed with only standard electroanatomic mapping systems, image integration using computed tomography, cardiac magnetic resonance imaging (CMR), intracardiac ultrasound, and fluoroscopic angiography is available and may increase the safety and efficacy of the procedure.^2,5–7^ Most major centers, clinical trials, and registries utilize some form of imaging; however, there is currently no consensus on the optimal modality.^2^ CMR can accurately map cardiac and pulmonary venous morphology without exposing patients to ionizing radiation.^8^ In addition, left ventricular functional analysis on dynamic CMR and late gadolinium enhancement (LGE) on gadolinium-enhanced CMR provides ancillary information that has demonstrated prognostic value in a variety of patient populations.^9–16^ and may have value in appropriate patient selection for PVAI.^17^ There are limited data suggesting that left ventricular (LV) LGE is more prevalent in patients with AF^18,19^ and Neilan et al recently demonstrated that LV LGE has strong prognostic value in this population. Accordingly, the purpose of the current study was to assess the incidence and pattern of LV LGE in a cohort of patients undergoing CMR before PVAI and compare the demographic and clinical characteristics of patients with and without LV LGE.

## MATERIALS AND METHODS

### Patient Population

The study was performed as a retrospective cross-sectional analysis using data from a larger, prospective study. The study protocol was approved by our Institutional Review Board and performed in accordance with HIPPA regulations. Inclusion criteria were all patients referred for PVAI for the treatment of symptomatic drug-refractory AF between July 19, 2011 and December 5, 2012; informed consent was obtained. Patients were excluded if they had contraindications to contrast-enhanced MRI (renal insufficiency as defined by an estimated glomerular filtration rate less than 60 mL/min, history of severe contrast allergy to contrast material, cardiac pacemaker, incompatible metallic implant). Sixty-eight consecutive patients were included who received CMR before PVAI between July 19, 2011 and December 5, 2012.

### Demographic and Clinical Data

Demographic and clinical data were acquired via review of electronic medical records at the time of initial enrollment. In addition to basic demographic information, cardiac risk factors, clinical cardiac history, and AF history were recorded. AF was categorized as paroxysmal (AF self-termination within 7 days), persistent (AF lasting >7 days or requiring cardioversion), or long-standing persistent (AF lasting >1 year). CHADS_2_ scores were calculated for each patient.^20^ LV ejection fraction (LVEF) was recorded as determined by transthoracic echocardiography performed in standard fashion at the time of initial evaluation.

### Imaging Protocol

Each patient underwent CMR (using 1.5-tesla Siemens Avanto or Siemens Aera; Siemens Healthcare; Erlangen, Germany) with the primary goal of left atrial mapping before PVAI. A 6-channel phased-array torso coil was used with a 6-channel spine matrix coil for a total of 12 independent receiver channels. LGE-CMR scans were acquired 15 to 20 minutes after injection of 0.2 mmol/kg of a gadolinium-based intravenous contrast material (gadopentetate dimeglumine; Bayer Healthcare Pharmaceuticals; Montville, NJ). LGE-CMR images were acquired with a 3-dimensional inversion recovery prepared fast spoiled gradient echo, respiratory navigated, electrocardiogram-gated, fat-suppressed sequence with the following parameters: typical TR 500–700 ms, field of view 340 mm, flip angle 10 degree, in-plane resolution 1.3 × 1.3 mm, and slice thickness 2.0 mm. The respiratory navigator was positioned on the right hemidiaphragm to limit respiratory gating artifacts. Trigger time for images was selected to correspond with left atrial diastole as determined on 4-chamber cine images. Navigator sequences average 7 to 10 minutes. The inversion time was identified for optimal nulling of the myocardium with a look-locker recovery (TI scout) scan and was typically between 280 and 300 ms, as previously reported.^21^. 30 msec was added to the optimal nulling time to account for gadolinium washout during the 3D LGE acquisition.

Standard steady-state free precession cine images were obtained in the horizontal long axis plane for each patient. Contiguous axial slices were acquired to cover the left ventricle and left atrium. The horizontal long-axis view is used in lieu of the short-axis view in our standard preatrial fibrillation CMR protocol for improved imaging of left atrial function.

### Image Analysis

**FIGURE 1:**
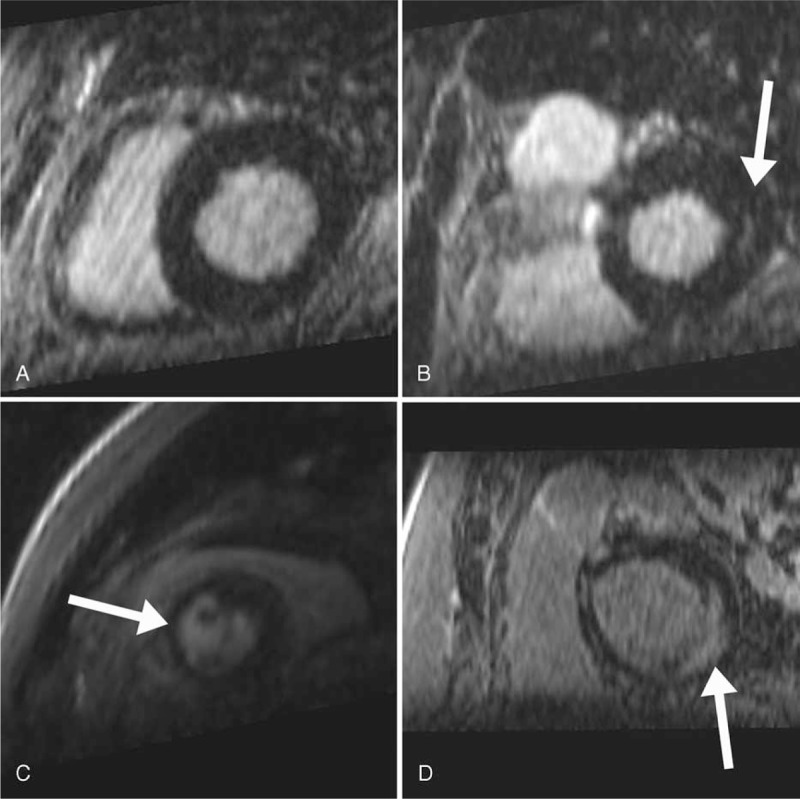
Examples of various grades of late gadolinium enhancement (LGE) (see text for further description): Panel A shows no LGE, panel B shows minimal LGE, panel C shows moderate LGE, and panel D shows severe LGE (arrows).

Two independent observers (JWN and SLZ) with 3 years and 4 years of cardiac imaging experience, respectively, analyzed the LGE and CMR cine images with disagreements resolved on consensus reading. Observers were blind to patients’ clinical and AF history. LV LGE was recorded on a per-patient (presence of any LGE) and per-segment basis using the American Heart Association 17-segment myocardial model. Each segment was visually characterized as having no LGE, minimal LGE (involving <25% of the myocardial segment), moderate LGE (involving 25%–50% of the segment), or severe LGE (>50% of the segment; Figure 1). Segments with LGE were further characterized as having subepicardial LGE, midwall LGE, or subendocardial LGE. Subendocardial LGE was stratified into groups according to the extent of LV wall involvement: <25%, 25%–<50%, 50%–<75%, 75%–<100%, or 100% (transmural). LV wall motion was subjectively evaluated on a per-patient basis and characterized as normal, global hypokinesis, or focal wall motion abnormality (WMA).

### Statistical Analysis

All statistical analyses were performed on commercially available dedicated statistics software (SPSS; SPSS Inc, Chicago, IL). The clinical characteristics of the study group, including demographic characteristics, cardiac risk factors, clinical cardiac history, and AF history, are described and stratified by patients with and patients without any LV LGE. For descriptive purposes, patients with LGE were further stratified by those with “expected/explained” LGE and those without expected LGE based on clinical history. Expected/explained patterns were defined as subendocardial LGE in patients with known prior myocardial infarction or amyloidosis, midmyocardial LGE in patients with hypertrophic cardiomyopathy, or any LGE in patients with sarcoidosis or myocarditis. Continuous variables are presented as mean (±SD) or median (interquartile range) and compared with the 2-sample *t* test if normally distributed or the Mann-Whitney test if data were nonparametric. Categorical variables are presented as frequency (percentage) and compared using the *χ*^2^ test. Multivariable logistic regression was used to examine the association of ventricular LGE with body mass index (BMI), persistent AF, and CHADS score. CHADS score was used because it is a readily available clinical risk stratification system that accounts for multiple cardiac risk factors (congestive heart failure, hypertension, age ≥75 years, diabetes mellitus, and history of stroke, transient ischemic attack, or thromboembolism), including several that showed significant or near-significant differences between patients with and without LV LGE on univariate comparative analysis. BMI and persistent AF were included because there were near-significant differences between patients with and without LV LGE. For the purposes of analysis, a CHADS score of 0 was used as reference and patients with scores above 0 were stratified into 2 groups: those with a CHADS score of 1 or ≥2. A *P* value <0.05 was considered statistically significant; all *P* values are 2-tailed.

## RESULTS

Six of 68 patients (9%) had nondiagnostic image quality on LGE-MRI sequences and were excluded from subsequent analysis, for a final cohort of 62 patients. Twenty-eight of 1054 (2.7%) LV segments were outside the field of view on LGE-MRI images and could not be evaluated. Two of 62 patients (3%; both without evidence of LGE) had nondiagnostic image quality on CMR cine images; these missing variables were considered missing-at-random and the patients were excluded from relevant analyses (ie, those involving LV WMA).

Clinical and demographic characteristics of the study population are presented in Table 1 Twenty-three of 62 (37%) patients were found to have LV LGE. Significantly, more patients with LGE had a history of hypertension or congestive heart failure. Significantly fewer patients with LGE had paroxysmal AF. Notably, there was no significant difference in LVEF (by echocardiography) between patients with and without LGE, and although a greater percentage of patients with LGE had focal or global WMA, the difference was not significantly significant (Table 1). In multivariable logistic regression, CHADS score ≥2 was independently associated with ventricular LGE (odds ratio [OR] 4.9, *P* = 0.039) after adjusting for BMI and persistent AF. There was also a trend for an association between persistent AF and LGE, which did not reach statistical significance (OR 3.0, *P* = 0.075).

**TABLE 1:**
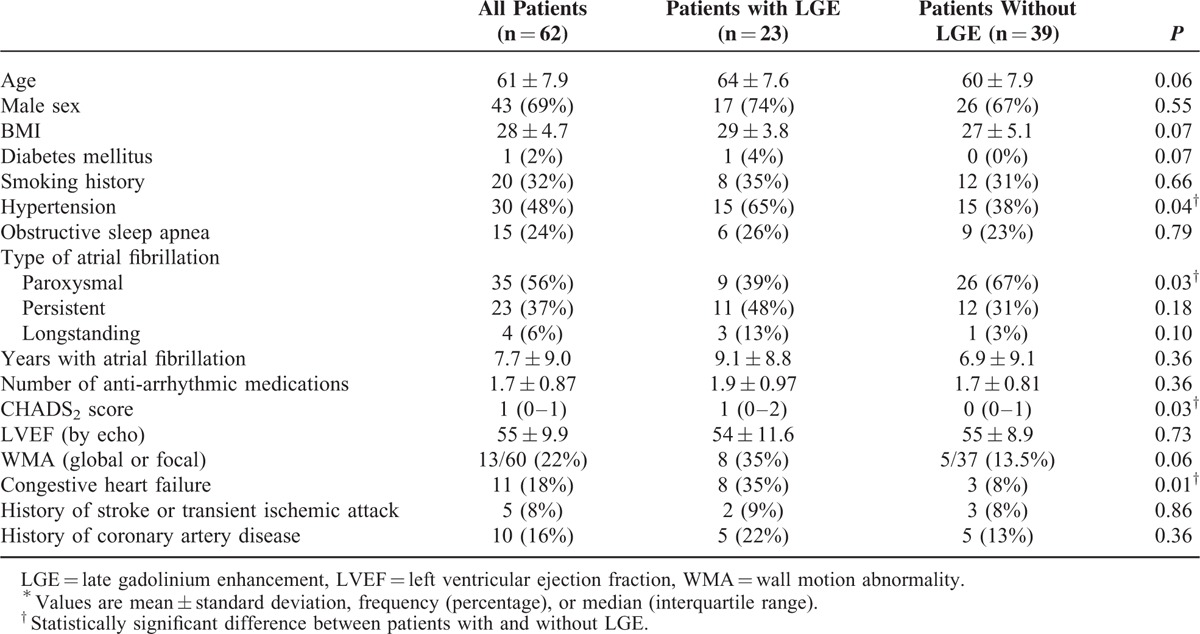
Clinical and Demographic Characteristics of Patients With and Without LGE^∗^

**FIGURE 2:**
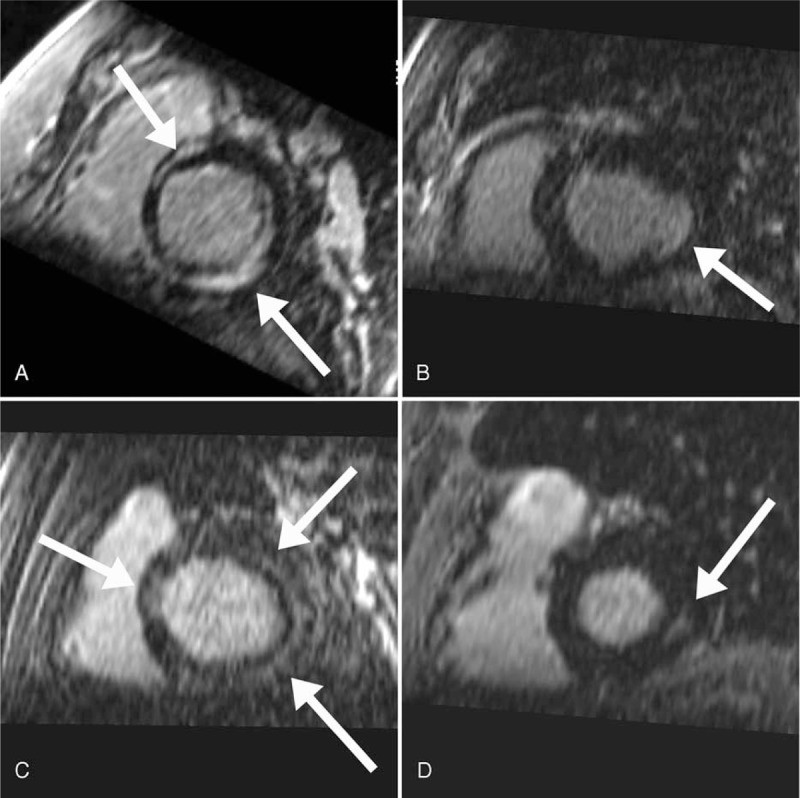
Short-axis images demonstrating various patterns of unsuspected late gadolinium enhancement (LGE) (arrows). Panel A shows extensive patchy basilar midmyocardial LGE in a 66-year-old man with no prior history of myocardial of coronary artery disease. Panel B shows an ischemic pattern of LGE (subendocardial) in a 73-year-old male without known coronary artery disease or history of infarction. Panel C shows near-circumferential basilar midmyocardial LGE in a 53-year-old male with history of nonischemic cardiomyopathy. Panel D shows the most common pattern of LGE in our cohort: inferolateral basilar midmyocardial LGE, seen in this 70-year-old male with a history of coronary artery disease but no know myocardial process.

**FIGURE 3:**
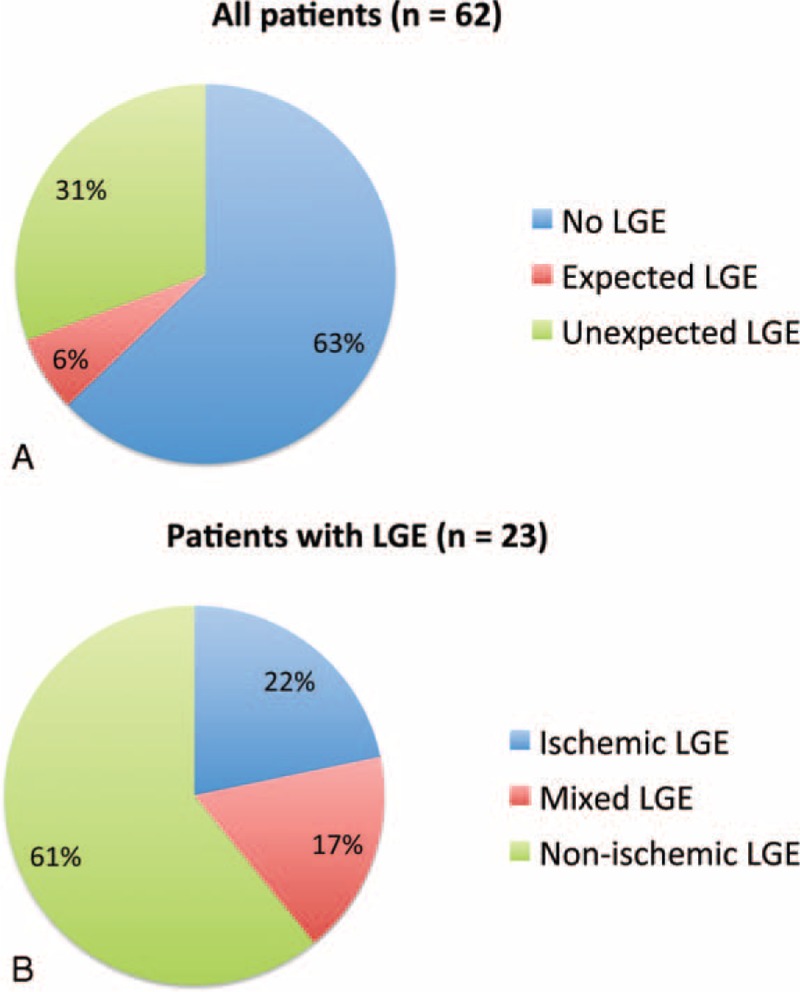
Pie charts demonstrating the percentage of the total patient population with expected and unexpected patterns of late gadolinium enhancement (LGE) (panel A) and the percentage of the subset of patients with LGE that had purely ischemic, purely nonischemic, or mixed ischemic and non-ischemic LGE patterns (panel B).

Table 2 summarizes the cardiac history and imaging patterns in the 23 patients that demonstrated LGE. The most common pattern was midmyocardial LGE (Figure 2), which was demonstrated in 17 of 23 patients (74%). Nine of 23 patients (39%) had evidence of subendocardial LGE suggestive of prior ischemia; of these patients, 3 had a history of coronary artery disease and only 1 had a known prior myocardial infarction. Four patients had both subendocardial LGE and LGE in a nonischemic pattern. Overall, 14 of 23 patients (61%) demonstrated purely nonischemic pattern LGE, 5 of 23 patients (22%) demonstrated purely ischemic pattern LGE, and 4 of 23 patients (17%) demonstrated a mixed ischemic and nonischemic pattern (Figure 3). Among all patients with LGE, an average of 3.0 ± 2.1 myocardial segments were affected. Extent of LGE was significantly higher in patients with a mixed pattern (5.5 ± 1.9 segments) compared with those with purely ischemic (2.6 ± 1.8 segments) or purely nonischemic (2.5 ± 1.8) patterns (*P* = 0.007), whereas there was no difference in extent between patients with purely ischemic versus purely nonischemic patterns (*P* = .92). The most common overall pattern of LGE was midwall involvement in the basal inferolateral LV myocardium (segment 5), which was seen in 11 patients (10/14 [71%] with a purely nonischemic pattern and 1/4 [25%] with a mixed pattern). Most (15/23; 65%) patients with LGE had no WMAs.

**TABLE 2:**
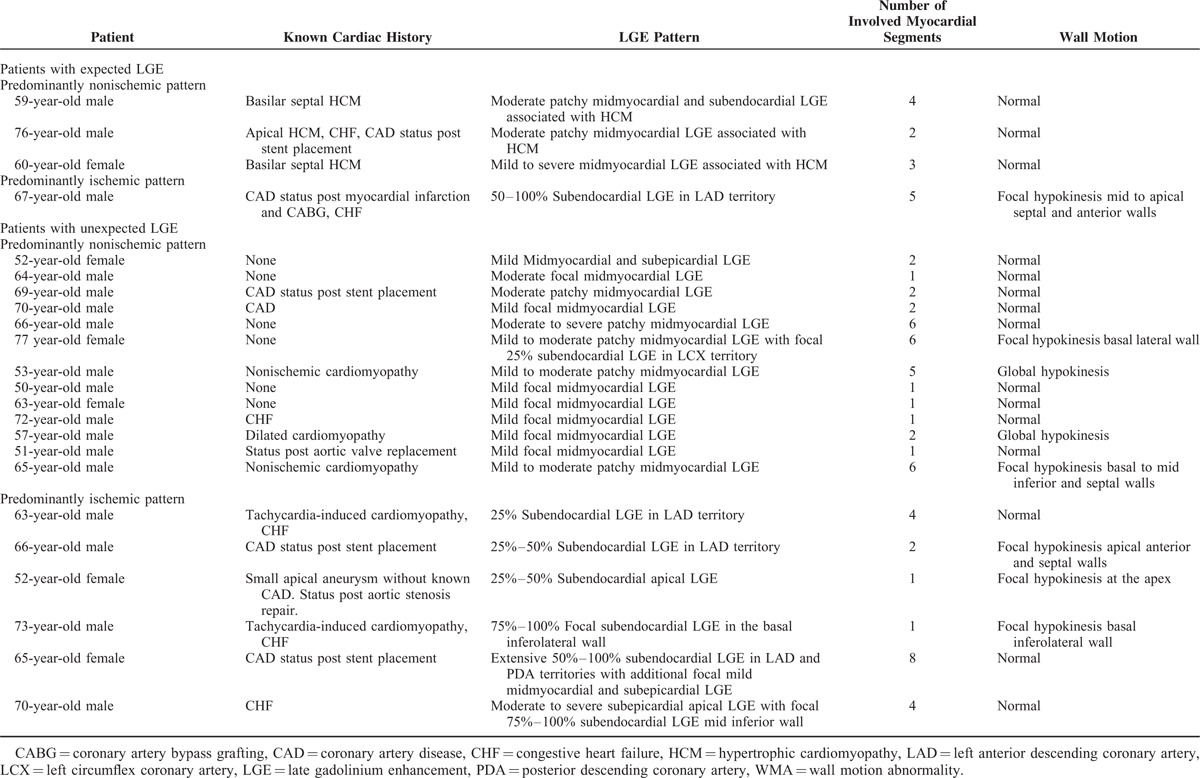
Cardiac History, LGE Pattern and Extent, and WMA in 23 Patients With LGE

Four of 23 patients (17%; Figure 3) had LGE in an “expected/explained” pattern based on clinical history – 3 with midmyocardial LGE associated with basilar (n = 2) or apical (n = 1) hypertrophic cardiomyopathy and 1 with subendocardial LGE and a known prior myocardial infarction. Of the remaining 19 patients, 4 had known congestive heart failure, 5 had known nonischemic cardiomyopathy, 4 had known coronary artery disease, and 2 had prior aortic valve repair or replacement. Six of 23 patients (26%) with LGE had no known coronary artery, valvular, or myocardial disease. There was no difference in the extent of LGE between patients with expected (3.5 ± 1.3 segments) and unexpected (2.9 ± 2.2 segments) LGE (*P* = 0.64).

## DISCUSSION

Our results show that LV LGE is common in patients with AF undergoing CMR before PVAI, with a prevalence of 37% in the current cohort. The incidence/pattern of LGE was unexpected in 31% of the entire study population and 83% of patients with LGE. A nonischemic pattern was most common, with 61% of patients demonstrating purely nonischemic LGE and 17% of patients demonstrating mixed ischemic and nonischemic LGE.

Several prior studies have suggested that patients with AF may have an increased prevalence of LGE. Papavassiliu et al^18^ examined a group of patients with hypertrophic cardiomyopathy and found that LGE was more commonly seen in AF patients. Recent data from the ROCKET AF trial showed that 17% of over 14,000 patients with AF had a history of prior myocardial infarction at enrollment, and those patients had a substantially higher risk of subsequent cardiac events.^22^ Likewise, Neilan et al recently examined the prevalence and prognostic value of LGE in 720 patients undergoing CMR before PVAI and demonstrated LGE in 15% of all patients and 13% of 664 patients without a clinical history or electrocardiographic evidence of myocardial infarction. LGE was found to be a strong predictor of mortality.^19^ Our population demonstrated a significantly higher prevalence of LGE at 37%. The discrepancy in overall LGE prevalence may be partially explained by the age of our cohort, which averaged 61 years compared with 56 years in Neilan et al's. Differences also could be in part due to chance, given our smaller sample size. Like our study, patients with LGE were significantly older compared with those without. Additionally, significantly more patients with LGE had a history of heart failure in Neilan et al's study, yet surprisingly, our population had a lower prevalence of heart failure (18% versus 26%).

Thirteen percent of our patients demonstrated clinically unexpected ischemic LGE, a higher percentage than Neilan et al's (6.6% when patients with a history of or electrocardiographic evidence for prior myocardial infarction were excluded). Again, this could be partially related to our older population, as other population-based studies with an older population have even higher rates of silent myocardial infarction (17% in one study of asymptomatic volunteers with median age 76 years).^23^ In addition, we used only clinical history to identify patients with prior myocardial infarction, and the additional utilization of electrocardiographic findings would be expected to eliminate some patients who are otherwise considered to have unexpected ischemic LGE. This pattern, which presumably represents silent myocardial infarction, has shown predictive value for future mortality^23,24^ and could alter patient management. Of note, there was no difference in LVEF between patients with and without LGE in our population and most patients with LGE had no WMA, indicating that the majority of LGE represented subclinical myocardial disease only detectable with CMR. Prior evidence has suggested that LGE predicts early mortality even with near-normal LVEF^24^ and the combination of these data makes a case for CMR utilization in this population.

Among those patients with LGE, a higher proportion of our patients demonstrated a nonischemic pattern compared with Neilan et al's, with a total of 78% of those with LGE showing pure nonischemic (61%) or mixed ischemic and nonischemic (17%) patterns. Although several patients had known hypertrophic cardiomyopathy and a compatible LGE pattern, the majority (79%) had unexpected LGE. Interestingly, the most commonly affected segment was the basal inferolateral wall, which was involved in 11 of 18 patients with mixed or purely nonischemic LGE patterns. This finding has been demonstrated in other populations with cardiac disease, including metabolic diseases (eg, Fabry's), sarcoidoisis, connective tissue disorders (eg, scleroderma), and infectious diseases (eg, Chagas)^25–27^; however, the underlying pathophysiology is unknown. Interestingly, this is also the most common area for detection of subepicardial LGE in myocarditis and muscular dystrophy and subendocardial LGE in patients with unrecognized myocardial infarctions.^28–30^ Some authors have proposed that this region of the myocardium is an area at risk for ischemia and remodeling because it is in a watershed vascular distribution between the right coronary artery and circumflex coronary artery territories.^28^ Similar to CMR-detected silent myocardial infarction, LGE in a nonischemic pattern has been shown to have prognostic value for predicting future mortality^9,13,31^ and could prompt initiation, alteration, or escalation of therapy when detected incidentally on pre-PVAI imaging.

Our study has several limitations. The cohort was small and the design retrospective. Our CMR protocol for PVAI assessment is limited and does not contain standard LGE and short-axis cine sequences for volumetric/functional analysis and LGE quantification. We did not evaluate the prognostic value of LGE, and we do not have data on the effects of CMR findings on subsequent patient management.

In conclusion, there is a high prevalence of unexpected LV scar in patients undergoing CMR before PVAI for AF, with most patients demonstrating a nonischemic pattern of LV LGE. In a smaller number of patients, silent infarctions were detected despite no clinical history of myocardial infarction. CHADS score ≥2 was an independent predictor of LGE. There was no difference in LVEF between patients with and without LGE, and most patients with LGE demonstrated no WMA, indicating that the majority of patients suffered from subclinical myocardial disease. Considering the prognostic value of LGE that has been previously demonstrated in a variety of patient populations, including those with AF, the high prevalence of LGE in our population may further argue for CMR as the modality of choice when imaging integration before PVAI is desired.
